# LncRNAs regulate metabolism in cancer

**DOI:** 10.7150/ijbs.40769

**Published:** 2020-02-10

**Authors:** Wenyu Lin, Qiyin Zhou, Chao-Qun Wang, Liyuan Zhu, Chao Bi, Shuzhen Zhang, Xian Wang, Hongchuan Jin

**Affiliations:** 1 Laboratory of Cancer Biology, Key Lab of Biotherapy in Zhejiang, Sir Run Run Shaw Hospital, Zhejiang University School of Medicine, Hangzhou 310016, Zhejiang, China; 2 Department of Medical Oncology, Sir Run Run Shaw Hospital, Zhejiang University School of Medicine, Hangzhou 310016, Zhejiang, China; 3 Department of Pathology, Affiliated Dongyang Hospital of Wenzhou Medical University, Dongyang 322100, Zhejiang, China; 4 Institute of Translational Medicine, Zhejiang University School of Medicine, Hangzhou 310029, Zhejiang, China; 5 Department of Obstetrics and Gynecology, Zhejiang Xiaoshan Hospital, Hangzhou 311201, Zhejiang, China

**Keywords:** Long non-coding RNA, Cancer, Metabolism

## Abstract

Metabolic reprogramming is a hallmark of cancer. Mammalian genome is characterized by pervasive transcription, generating abundant non-coding RNAs (ncRNAs). Long non-coding RNAs (lncRNAs) are freshly discovered functional ncRNAs exerting extensive regulatory impact through diverse mechanisms. Emerging studies have revealed widespread roles of lncRNAs in the regulation of various cellular activities, including metabolic pathways. In this review, we summarize the latest advances regarding the regulatory roles of lncRNAs in cancer metabolism, particularly their roles in mitochondrial function, glucose, glutamine, and lipid metabolism. Moreover, we discuss the clinical application and challenges of targeting lncRNAs in cancer metabolism. Understanding the complex and special behavior of lncRNAs will allow a better depiction of cancer metabolic networks and permit the development of lncRNA-based clinical therapies by targeting cancer metabolism.

## Introduction

Cancer cells can reprogram metabolism to support them with sufficient building blocks, appropriate redox status and rapid ATP generation in favor of survival, growth or metastasis. For a long time, metabolic reprogramming has been regarded as a hallmark of cancer 1. Reprogrammed cancer metabolism mainly includes changes of three classes of molecules including carbohydrates, lipids, and amino acids, eventually altering mitochondrial function, glycolysis, glutaminolysis, and lipid metabolism 2. For example, cancer cells exhibit high rate of aerobic glycolysis, which was known as Warburg effect 3, 4. Accumulating evidence indicates that cancer metabolism can be regulated by signaling pathways involved in cell growth and proliferation 5-8. These studies have expanded our understanding of mechanisms underlying cancer metabolic reprogramming and provided insights into clinical diagnosis, prognosis, and treatment. However, the multifaceted cancer metabolism still needs further investigations.

Among the genome transcribed, less than 2% of the genome are protein-coding genes, while more than 90% of the rest are transcribed into ncRNAs 9. According to the size, ncRNAs can be subdivided into small ncRNAs such as miRNAs, small nucleolar RNAs (snoRNAs), PIWI-interacting RNAs (piRNAs) and transfer RNA-derived small RNAs (tsRNAs), as well as the newly characterized lncRNA class. LncRNAs are large RNA transcripts longer than 200 nucleotides with no or limited protein-coding potential and limited evolutionary conservation 10. Among genes expressed in human transcriptome, over 68% are transcribed into lncRNAs. Based on the locations relative to protein-coding genes, lncRNAs are classified into: (1) sense, or (2) antisense that cover exons of the nearest transcripts in the same or opposite direction; (3) bidirectional, that the initial transcription sites of lncRNA and the protein-coding gene on the opposite strand are closely localized, (4) intronic, that the whole lncRNA transcript localizes within the intron of a second transcript, and (5) intergenic, that set within the genomic interval of two genes^11^.

LncRNAs have been found to involve in various physiological and pathological cellular activities, such as adipogenesis, inflammation, cell differentiation and tumorigenesis, via genomic expression modulation, epigenetic modification and post-transcriptional regulation in *cis* or in *trans* by interacting with chromatins, proteins and RNAs in the nucleus or cytoplasm 12-19. In the nucleus, lncRNAs can modify gene expression by directly interacting with DNA or chromatin regulators, such as transcription factors and RNA binding proteins, acting as enhancers, decoys, scaffolds or guides. While in the cytoplasm, lncRNAs enable mRNA decay, modulate the stability or translation of mRNAs, compete with microRNA for binding to mRNA and can be processed into microRNAs 20. Nowadays, lncRNAs are increasingly drawing attention and flourishing evidence has warranted lncRNAs associate with multiple diseases, notably cancer 21. In cancer cells, lncRNAs are aberrantly expressed as classical oncogenes or tumor suppressors and correlate with the altered metabolism 1, 22. Therefore, targeting lncRNAs promises great potential as an alternative and workable therapy for cancer in the context of aberrant metabolism. In this review, we focus on metabolism-related lncRNAs and discuss their regulatory roles in cancer metabolism as well as their potential clinical translation via the regulation of cancer metabolism.

## LncRNAs regulate mitochondrial function

Mitochondria are the center of many biochemical processes including oxidative phosphorylation (OXPHOS), the krebs cycle, intracellular calcium balance, and the synthesis of cytosolic biosynthetic precursors such as amino acids, nucleotides, lipids, heme and NADPH 23, 24. As mitochondria play an essential role in multiple cellular biological processes, proper mitochondrial quality control and component integrity is pivotal for cancer maintenance and progression, particularly cancer cell metabolism.

Mitochondria are highly dynamic organelles associated with constant fusion and fission, which affect mitochondria shape, distribution and function 25, 26. Mitochondrial dynamics has been found to be involved in cell metabolism 27-30. For instance, disruption of mitochondrial fusion proteins MFNs or OPA1 causes reversible cellular respiration defects, while downregulation of mitochondrial fission protein DRP1 attenuates mitochondrial respiratory capacity 30, 31. Moreover, mitochondrial morphology could be adjusted by mitochondrial dynamics change in response to nutrient availability. For example, cancer cells dominant in OXPHOS activity tend to have condensed mitochondria, while those dependent on glycolysis show more orthodox conformation of mitochondria 32. Thus, exploring the roles lncRNAs play in mitochondria dynamics is of great importance to understand cancer metabolism.

**Figure 1:**
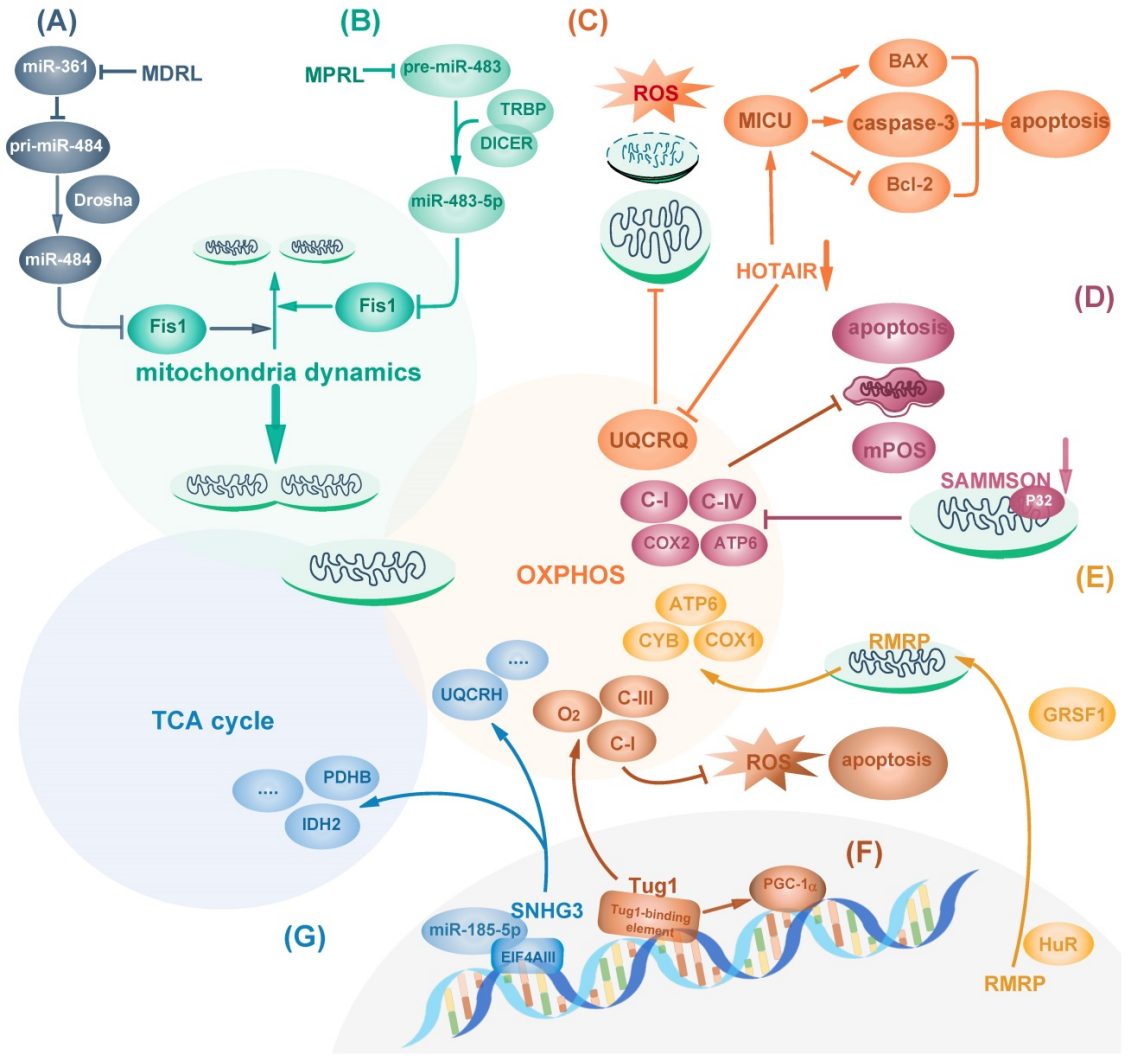
LncRNAs regulate mitochondria function. (A, B) LncRNA MDRL (A) and MPRL (B) act as sponges of miR-484 and miR-483-5p, respectively, which targets Fis1 to inhibit its expression and regulate mitochondrial dynamics. (C) HOTAIR knockdown impairs mitochondrial function via reducing its OXPHOS components including UQCRQ and triggers ROS stress. Also, HOTAIR blockage activates MICU and induces apoptosis by reducing Bcl-2 and increasing caspase 3 and BAX. (D) Mitochondrial part of SAMMSON inhibits mitochondrial membrane potential depolarization, mPOS and tumor apoptosis by interacting with p32 and enhancing its function. (E) Nuclear encoded RMRP transports into mitochondria facilitated by HuR and GRSF1 to induce mtDNA replication and increase OXPHOS subunit, thus promoting mitochondrial respiration. (F) Tug1 interacts with tug1-binding element upstream of PGC-1α promoter to maintain complex I and III activity and increase mitochondrial bioenergetics. (G) SNHG3 sponges miRNA-186-5p and interacts with EIF4AIII to regulate gene expression related to TCA cycle and OXPHOS activity such as PDHB, IDH2 and UQCRH.

Recently, studies have shown that nuclear-encoded lncRNAs regulate mitochondrial dynamics. FIS1 localizes on mitochondrial outer membrane and acts as a receptor for DRP1 recruitment to promote mitochondrial fragmentation, and FIS1 can induce cytochrome c dependent apoptosis 33. Mitochondrial dynamic related lncRNA (MDRL) downregulates miR-361 expression and indirectly upregulates miR-484, a negative regulator of Fis1 protein translation, by reducing the interaction between miR-361 and pri-miR-484. MiR-361 can bind to pri-miR-484 and inhibit its maturation by Drosha into pre-miR-484 in the nucleus, resulting in increased Fis1 and apoptosis. Thus, MDRL indirectly regulates mitochondrial fission and apoptosis 34, 35 (Fig. 1A). In contrast, focally amplified lncRNA on chromosome 1 (FAL1) inhibits apoptosis and cytochrome c release in esophageal squamous cell carcinoma (ESCC) cells by suppressing DRP1, which promotes cancer cell survival and increases mitochondrial respiration 36. Fis1 can also be targeted by other microRNAs such as miR-483-5p. In tongue squamous cell carcinoma (TSCC), miRNA processing-related lncRNA (MPRL) can upregulate Fis1 by preventing its upstream regulator miR-483-5p generation from TRBP-DICER-complex mediated recognition and subsequent cleavage of pre-miR-483. As the result, MPRL overexpression increases FIS1 expression to promote mitochondrial fission, inhibit tumor growth and enhance cisplatin sensitivity 37 (Fig. 1B). Though no evidence so far has directly pointed out how lncRNAs regulate cancer cell metabolism by affecting mitochondrial dynamics, it is rational to further investigate into the prospective correlations.

In addition to mitochondrial dynamics, mitochondrial contents related to OXPHOS can also be targeted by lncRNAs in diverse manners. LncRNAs can regulate cell metabolism and cancer cell survival by affecting mitochondria components including complexes I-IV and other subunits such as ATPase on mitochondria inner membrane 38. Hox Transcript Antisense Intergenic RNA (HOTAIR), one of the few well-studied lncRNAs, is a negative prognostic indicator of many cancers such as pancreatic cancer 39. In Hela cells, HOTAIR knockdown causes mitochondria dysfunction including reduced expression of OXPHOS component Ubiquinol-Cytochrome C Reductase Complex III Subunit VII (UQCRQ) and impairment of OXPHOS activity. Therefore, it results in increased intracellular ROS stress, vacuoles formation, mitochondrial morphology changes shown as swollen with loss of cristae, and mitochondria deprivation 40. Mitochondrial calcium uptake 1(MICU1) is a core component of mitochondrial calcium uniporter complex for regulating calcium release and maintaining proper mitochondrial membrane potential 41. HOTAIR blockage in head and neck squamous cell carcinoma (HNSCC) activates MICU1-dependent mitochondria-related apoptosis by reducing Bcl-2 and increasing pro-apoptotic proteins including BAX 42 (Fig. 1C). However, it remains unknown how HOTAIR regulates mitochondria functions. In addition to HOTAIR, SAMMSON, which primarily locates in the cytoplasm and mitochondria, is lincRNA specific for melanoma survival independent of MITF or BRAF, NRAS and p53 status, moreover, it can regulate mitochondria function 43. SAMMSON silencing causes mitochondria structure aberration and decreases the activity of respiratory complexes I and IV, leading to mitochondrial membrane potential depolarization, mitochondrial precursor-over-accumulation stress (mPOS) and cell apoptosis in melanoma. Mechanistically, the mitochondria part of SAMMSON directly interacts with p32, a protein required for mitochondrial 16S rRNA maturation, to enhance its function 44. As a result, its silencing decreases mitochondrial fraction of p32 to reduce mtDNA-encoded COX2 and ATP6, but not nuclear-encoded SDHA and NDUFS3 or other ETC components, thus inducing fragmented mitochondria cristae and less mitochondria matrix 44 (Fig. 1D). Similarly, lncRNA RMRP can also affect mtDNA-encoded proteins. RMRP is encoded by nuclear DNA and is transported into mitochondria matrix by RNA-binding proteins HuR and GRSF1. RMRP induces mtDNA replication and increases OXPHOS subunit ATP6, COX1 and CYB, which facilitate mitochondrial respiration in Hela cells 45 (Fig. 1E).

Besides being regulated in the cytoplasm, mitochondrial dynamics and function can be modulated from the nucleus. LncRNA nuclear enriched abundant transcript 1 (NEAT1) forms the main structural component of paraspeckles which can mediate the retention of nuclear-encoded mRNA. The middle region of NEAT1_2, one of NEAT1 isoform, is in the core region, surrounded by its 5' and 3' ends and another isoform NEAT1_1 46. Upon mitochondrial protein depletion or mito-stressor induction, nuclear transcription factor ATF2 binds to the promoter of NEAT1 and enhances the transcription of NEAT1 long-isoform NEAT1_2, promoting the elongation of paraspeckles. Elongated paraspeckles enhance the retention of nuclear-encoded mito-mRNAs which encodes proteins involved in mitochondrial function, including cytochrome c, subunit of NADH dehydrogenases and Carnitine O-palmitoyltransferase 1 47. Thus, NEAT1 exerts important effect on the regulation of mitochondrial homeostasis. Moreover, NEAT1 depletion leads to mitochondria elongation by inhibiting mitochondrial fission via decreased DRP1 expression and phosphorylation, causing mitochondrial dysfunction including reduction in mtDNA, respiration capacity, ATP output, extracellular acidification rate (ECAR) and reduced cell proliferation 47.

Some transcription factors essential for the transcription of mitochondrial proteins can also be regulated by lncRNAs. Peroxisome proliferator-activated receptor-c coactivator-1α (PGC-1α), one central transcriptional coactivator of genes related to mitochondrial biogenesis and mitochondrial oxidative metabolism, is dysregulated in several cancer types including colorectal cancer 48. LncRNA taurine-upregulated 1 (Tug1) interacts with tug1-binding element upstream of PGC-1α promotor, triggering PGC-1α transcription and improving mitochondrial bioenergetics. Tug1 knockdown inhibits OXPHOS subunit complex I and III activity, decreases ATP production, increases intracellular ROS and induces apoptosis, which can be rescued by overexpressing PGC-1α 49 (Fig. 1F).

In addition to gene transcription, protein translation can be targeted by lncRNAs to affect cancer metabolism and mitochondrial function. Eukaryotic translation initiation factor 4AIII (eIF4AIII) is an ATPase and RNA helicase that mediates pre-mRNA splicing, translation inhibition and nonsense-mediated decay in the nucleus 50, 51. eIF4AIII has target sites at mRNAs of metabolic enzymes involved in TCA cycle and oxidative phosphorylation, such as PKM, IDH2 and UQCRH. LncRNA SNHG3 is predicted to bind to and inhibit eIF4AIII to favor cancer cell survival. Moreover, SNHG3 sponges miR-186-5p to inhibit its binding to 3'UTR of PDHB 52 (Fig. 1G).

Collectively, lncRNAs are unprecedented essential regulators of mitochondrial function and metabolism by modifying mitochondrial dynamics and respiration. These regulatory operations exert great impact on cellular response to oncogenic stimuli and cancer cell behaviors.

## LncRNAs regulate glucose metabolism

**Figure 2:**
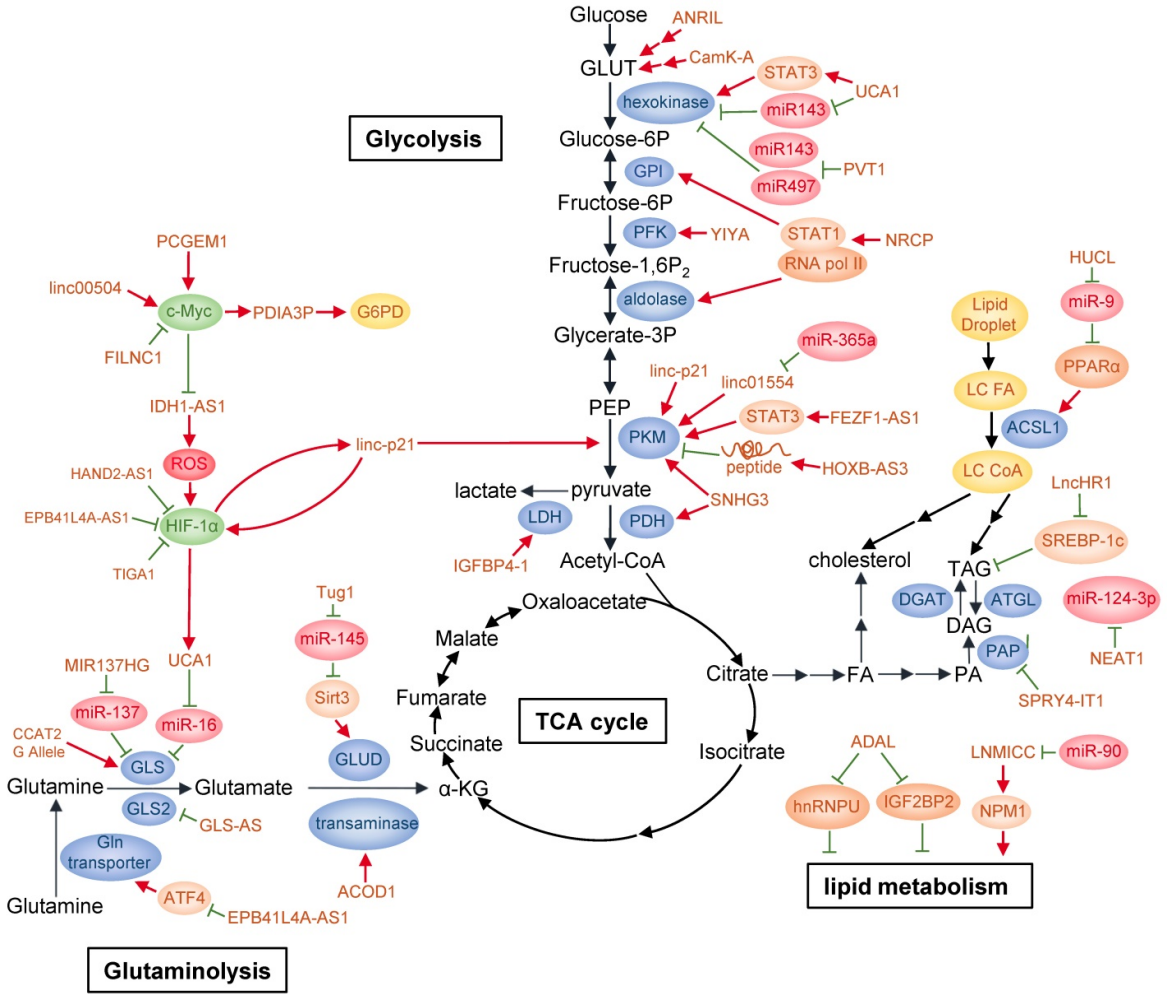
LncRNAs regulate cancer metabolism. By regulating glycolysis, glutaminolysis, and lipid metabolism, lncRNAs can reprogram major cancer metabolism pathways. Detailed mechanisms of action of these lncRNAs are described in the text.

Glucose is the major carbon source for cellular biosynthesis and energy generation, and altered glucose metabolism is one of the first identified hallmarks of cancer 3. Glucose transporters and multiple enzymes involved in or associated with glucose metabolism in cancers are found to be targeted by lncRNAs through diverse mechanisms (Fig. 2).

To date, many lncRNAs have been reported to be regulated by several well-known transcription factors in response of glucose availability in the environment or serving as mediators to modulate glucose metabolism. Among them, p53 is a frequently mutated or deleted tumor suppressor in various tumors. A number of studies have shown that p53 is closely correlated with cancer metabolism covering glycolysis, oxidative phosphorylation and pentose-phosphate pathway (PPP) 53-55. p53 can activate the transcription of many lncRNAs in addition to well-known mRNAs. It has been found that p53 directly upregulates lncRNA Tp53-regulated inhibitor of necrosis (TRINGS) in multiple tumor cells after glucose deprivation. TRINGS facilitates tumor growth* in vitro* and *in vivo* by binding to STRAP and inhibiting STRAP-GSK3β-NF-κB necrotic pathway 56. LincRNA-p21, a downstream transcriptional target of p53, can be upregulated upon DNA damage to repress the transcription of p53 targeted genes 57. LincRNA-p21 knockdown upregulates PKM2 expression through PTEN/AKT/mTOR pathway, thus activating glycolysis and enabling prostate cancer progression 58. Moreover, lincRNA-p21 is upregulated by HIF-1α and reciprocally binds to HIF-1α, preventing HIF-1α from degradation by interrupting HIF-1α-VHL interaction. Therefore, it can promote HIF-1α accumulation and hypoxia-enhanced glycolysis 59.

C-Myc is another oncogenic transcription factor upregulated in a wide variety of cancers. It directly or indirectly targets genes involved in glycolysis, including LDHA, GLUT1, HK2, PFKM, and ENO 60. LncRNA prostate cancer gene expression marker 1 (PCGEM1) is specifically expressed in prostate cancer and can be induced by androgen. PCGEM1 acts as a coactivator of c-Myc and facilitates c-Myc as well as other regulators to transcriptionally activate PCGEM1-targeted metabolic genes by increasing histone H3 and H4 acetylation. Besides, PCGEM1 knockdown downregulates various metabolic genes and inhibits glycolysis via unknown transcription factors, revealing another layer of metabolic regulation independent of androgen signaling 61. In colon cancer, linc00504 could promote cell progression both *in vivo* and* in vitro* by regulating multiple metabolic pathways, including glucose metabolism, at transcriptional level. It interacts with c-Myc to promote its chromatin recruitment and enhance its transactivation activity 62. Recently, FoxO-induced long non-coding RNA 1 (FILNC1) has been reported to repress the transcription of glycolysis genes, including glucose transporter 1 and 3 (GLUT1/3), hexokinase 2 (HK2), aldolase C (ALDOC), lactate transporter MCT4 as well as pyruvate dehydrogenase kinase 1 (PDK1) and PDK4. In detail, FILNC1 suppresses the translation of c-Myc mRNA by sequestering AUF1, which is further enhanced under glucose starvation. Thus, FILNC1 downregulation enhances Warburg effect and indicates poor prognosis in renal cell carcinoma 63.

In addition, lncRNAs can activate the transcription of metabolic enzymes directly or indirectly. For example, in bladder cancer, lncRNA urothelial carcinoma associated 1 (UCA1) is transcriptionally upregulated by HIF-1α under hypoxia 64. It can stimulate glycolysis indirectly through UCA1-mTOR-STAT3/miR143-HK2 cascades, in which STAT3 transcriptionally upregulates HK2 mRNA level and miR143 reduction increases hexokinase 2 (HK2) protein level 65. Antisense non-coding RNA at the INK4 locus (ANRIL) promotes AML progression by regulating adiponectin receptor1 (AdipoR1)/AMPKα/SIRT1 pathway, elevating GLUT1 and LDHA expression to promote AML cell survival 66. Under glucose deprivation, lncRNA insulin-like growth factor binding protein 4-1 (IGFBP4-1) induces ATP production and increases expression of HK2, PDK1 and LDHA at transcriptional level to promote glucose metabolism and tumor progression in lung cancer, though the detailed molecular mechanism was not investigated 67. Besides its role in OXPHOS mentioned above, SNHG3 also regulates glycolysis and TCA cycle in ovarian cancer at transcriptional level by targeting genes such as pyruvate kinase M (PKM), pyruvate dehydrogenase E1 component subunit beta (PDHB) and isocitrate dehydrogenase 2 (IDH2) 52. Moreover, ceruloplasmin (NRCP), a lncRNA significantly overexpressed in ovarian cancer, increases the interaction between RNA polymerase II and STAT1 in the nucleus, which enhances the transcription of enzymes involved in glycolysis such as glucose-6-phosphate isomerase (GPI), ALDOA and ALDOC 68. In a siRNA library screening, lncRNA for calcium-dependent kinase activity (CamK-A) was found to affect glycolysis and cell survival in various cancer types. In response to hypoxia, increased cytosolic calcium flux from endoplasm reticulum induces CamK-A upregulation. CamK-A activates pregnancy upregulated non-ubiquitously expressed CaM kinase (PNCK) and phosphorylates IĸBα, subsequently activating the transcription of genes downstream of NF-ĸB, including GLUT3 for enhanced glucose uptake and cytokines for tumor microenvironment remodeling such as microphage infiltration and angiogenesis 69. LncRNA metastasis-associated lung adenocarcinoma transcript 1 (MALAT1) modulates both gluconeogenic and glycolytic genes including GLUT1, HK2, ENO1 and PKM2 transcriptionally by translationally increasing transcription factor TCF7L2 protein level in a mTORC1-4EBP1 axis mediated cap-dependent manner 70.

Accumulating evidence has indicated that lncRNAs can act as competing endogenous RNAs (ceRNAs) to regulate glucose metabolism. LncRNA H19 was previously reported to be oncogenic in glioma, hepatocellular carcinoma (HCC) and bladder cancer, but none of them were related to cancer metabolism 71-74. A recent study found that lncRNA H19 functions as a ceRNA of miR-106a-5p and competitively binds to E2F3, a transcription factor facilitating the expression of glucose metabolic genes, thus promoting glucose metabolism in melanoma and serving as an indicator of poor prognosis 75. Upon glucose starvation, lncRNA Heart and Neural Crest Derivatives Expressed 2-antisense 1 (HAND2-AS1) downregulation significantly induces glycolysis, decreases apoptosis as well as increases cell viability and colony formation in osteosarcoma. Acting as a ceRNA, HAND2-AS1 facilitates fructose-1, 6-bisphosphatase 1 (FBP1) bind to HIF-1α mRNA, which decreases HIF-1α mRNA level and inhibits the transcription of metabolic genes such as GLUT1, GLUT3, HK2, ALDOC and MCT4 downstream of HIF-1α 76. In osteosarcoma, lncRNA PVT1 induces glycolysis via inhibiting the negative regulation of HK2 mRNA by miR-497 77. In gallbladder cancer (GBC), PVT1 positively regulates HK2 expression by sponging miR-143 78. Therefore, PVT1 regulates glycolysis through competing with different microRNAs in a cancer type-specific manner.

LncRNAs also regulate glycolytic enzyme function by affecting protein modification. YIYA or linc00538 increases 6-phosphofructo-2-kinase/fructose-2, 6-biphosphatase 3 (PFKFB3) enzymatic activity through CDK6-dependent phosphorylation, thereby enhancing the conversion of G6P/F6P to FBP/GBP. Increased expression of YIYA and CDK6 maintain elevated glycolysis in breast cancer, thus disfavoring patients' survival 79. LncRNA IDH1 antisense RNA1 (IDH1-AS1) enhances IDH1 enzymatic activity at post-translational level by facilitating IDH1 homodimerization, resulting in increased production of α-ketoglutarate (α-KG) as well as downregulation of ROS 80, 81. Under hypoxia, PHDs catalyzes HIF1α hydroxylation, a prerequisite for its conjugation to von Hippel-Lindau (VHL) and subsequent degradation through ubiquitin-proteasome pathway 82. However, the hydroxylaton of HIF-1α could also be regulated under normoxia by variant stimulants, such as TCA cycle intermediates α-ketoglutarate, succinate, fumarate and malate 81. Thus, IDH1-AS1 can indirectly activate HIF-1α by increased α-KG under normoxia. IDH1-AS1 is also transcriptionally repressed by c-Myc 80. This c-Myc- (IDH1-AS1)-IDH1-αKG/ROS-HIF1α axis links two of the most important cancer metabolism effectors together, indicating the complexity of metabolic regulation network.

LncRNAs can also affect glycolytic enzyme degradation. LINC01554, negatively regulated by miR-365a in HCC and correlated with poor clinical outcomes, suppresses aerobic glycolysis by decreasing PKM2 level through ubiquitin-proteasome pathway and inhibiting Akt/mTOR pathway in the cytoplasm 83. FEZF1-AS1 promotes cancer maintenance and metastasis in colorectal cancer by binding to and increasing PKM2 abundance through transcription in the nucleus, or protecting PKM2 from degradation in proteasome pathway in the cytoplasm. In the nucleus, upregulated PKM2 activates STAT3 pathway, which further enhances PKM transcription, while accumulated cytoplasmic PKM2 enables increased PKM2 tetramer formation, which promotes pyruvate production from phosphoenolpyruvate (PEP) 84.

As indicated by the name, most lncRNAs do not encode peptides 85. However, this concept was overturned in recent studies. For example, lncRNA HOXB cluster antisense RNA 3 (HOXB-AS3) encodes a 53-aa peptide, which suppresses colorectal cancer (CRC) progression. Mechanistically, the HOXB-AS3 peptide reduces PKM2 transcription by selectively binding to hnRNP A1 binding domain directed at the sequences flanking PKM exon 9. Thus, lactate production is reduced and glucose metabolism is suppressed. Consistently, colon cancer tissues of various stages conformably show decreased mRNA and protein levels of HOXB-AS3 86.

Pentose phosphate pathway, bifurcated from glycolysis, is an important source of intracellular NADPH and ribonucleotides through oxidation, and pentose phosphates by non-oxidation, which cope with oxidative stress and supply materials for nucleic acid and fatty acid synthesis 87. PPP has been reported to be dysregulated in cancers to favor proliferation and metastasis, and is regulated by multiple oncogenes such as TP53 and mTORC1 88, 89. Glucose-6-phosphate dehydrogenase (G6PD) is a rate-limiting enzyme that catalyzes dehydrogenation of glucose-6-phosphate (G6P) into NADPH and 6-phosphogluconolactone. G6PD expression is transcriptionally activated by c-Myc, which is further enhanced by lncRNA protein disulfide isomerase family A member 3 pseudogene 1 (PDIA3P) via interacting with c-Myc. Thus, PDIA3P promotes PPP and enables multiple myeloma progression 90. Another study showed that lncRNA prostate cancer gene expression marker 1 (PCGEM1) binds to target promoters through interacting with c-Myc and increasing its chromatin recruitment, thus enhances c-Myc transactivation activity and affects multiple metabolism pathways including PPP 61.

Targeting enhanced glucose metabolism in cancer has already been clinically investigated to develop therapeutic drugs or available diagnostic tools. The various aspects of lncRNAs involved in glucose metabolism denote a vital way to further the insight into cancer progression. Therefore, lncRNAs possess great potential in developing effective therapies and diagnostic approaches against dysregulated glucose metabolism in cancer.

## LncRNAs regulate glutamine metabolism

Pyruvate influx into mitochondria is the initial step to elicit TCA cycle. In cancer cells, the transportation of pyruvate into mitochondria is drastically decreased due to increased lactate production. To maintain mitochondrial function and essential TCA cycle that supply macromolecules required for cancer cell proliferation, metabolic intermediates flowing into the mitochondria need replenishment from alternative sources. Glutamine, the most abundant amino acid in human body, is involved in most biosynthetic and bioenergetic pathways in many cancer cells. In this way, glutamine serves as an important carbon or nitrogen source through mitochondrial anaplerosis 91. Once transported into the cytoplasm, glutamine is catalyzed into glutamate by glutaminase (GLS1/GLS2), then converted into α-KG, an intermediate in TCA cycle, via glutamate dehydrogenase (GLUD/GDH) or transaminases, coupled with production of NADH, NADPH, ammonium, and other nonessential amino acids (NEAAs). Thus, glutamine provides precursors, such as citrate and oxaloacetate, for the synthesis of amino acids, nucleotides and fatty acids. Glutamine also donates amide (γ-nitrogen) group for NEAAs, purine and pyrimidine synthesis 91, 92. Besides, glutamine is an important source of glutathione, a dominant factor for relieving intracellular redox stress. Therefore, glutamine plays a widespread role in cellular activity, and lncRNAs also aim at several enzymes in glutamine metabolism (Fig. 2).

Glutaminase is the rate-limiting enzyme in glutaminolysis and is subjected to fine-tuned regulation. It is generated mainly from GLS1 and the two isoforms glutaminase isoform C (GAC) and glutaminase kidney isoform (KGA) differ in their catalytic ability. LncRNA Colon Cancer-Associated Transcript 2 (CCAT2) interacts with cleavage Factor I (CFIm) subunits in an allele-specific manner. The G allele of CCAT2 at rs6983267 SNP site preferentially binds to CFIm25 rather than CFIm68. The former complex interacts with GLS pre-mRNA and leads to increased splicing of GLS mRNA into GAC, resulting in increased glutamine metabolism. Cancer cell with CCAT2 G allele display more activated cellular metabolism of glucose, TCA cycle, glutamine and fatty acids. As a result, rs6983267 SNP with G allele, rather than T allele, is associated with greater risk in colorectal cancer 93. Heat Shock Factor 1 (HSF1) can recruit DNMT3a to methylate the promoter of lncRNA MIR137HG and increase its transcription, which subsequently represses the expression of miR-137. GLS1 mRNA can be targeted and suppressed by miR-137. As a result, HSF1 stimulates GLS1-dependent glutaminolysis and activates mTOR to promote colorectal carcinogenesis 94. Besides GLS1, GLS2 is also related to reprogrammed glutaminase activity in cancer. LncRNA urothelial carcinoma-associated 1 (UCA1) interferes the negative regulation of GLS2 mRNA by miR-16, upregulating GLS2 expression to promote glutaminolysis, inhibit ROS production and protect cells from oxidative toxicity in bladder cancer 95-97.

GLS-AS is a nucleus-located antisense lncRNA of GLS. In pancreatic cancer cells, upon glucose and glutamine deprivation, GLS-AS is downregulated by c-Myc at transcriptional level and reduces its binding to GLS pre-mRNA, which inhibits GLS pre-mRNA degradation via ADAR/Dicer-dependent RNA interference. Thus, GLS is upregulated at post-transcriptional level and promotes pancreatic cancer cell survival as well as metastasis. Conversely, c-Myc protein stability is impaired partially in proteasome pathway by GLS-AS in a GLS-dependent manner, hence forming a negative feedback loop between c-Myc and GLS-AS 98.

Several studies have shown that transaminases are regulated by lncRNAs. A special viral infection-induced lncRNA aconitate decarboxylase 1 (ACOD1) directly interacts with glutamic-oxaloacetic transaminase-2 (GOT2) to enhance its enzymatic activity and provide metabolites for viral replication 99. ACOD1 expands lncRNA function in response to various stimulants, indicating that lncRNAs are involved in a wide regulatory network. Given that several types of cancer including HCC are associated with viral infection, the regulatory role of lncRNA found in viral infection may provide a new therapeutic target for such cancers. Another study showed that lncRNA taurine upregulated gene 1 (Tug1) prevents miR-145 mediated degradation of Sirt3 mRNA, promoting Sirt3 protein expression. Sirt3 positively regulates GDH translation and enhances glutamine consumption, α-KG and ATP production in intrahepatic cholangiocarcinoma (ICC) 100. Thus, Tug1 indirectly upregulates GDH to assist glutamine metabolism.

Studies also reveal that lncRNAs can regulate the transcription of intermediates in glutamine metabolism or other energetic metabolism through epigenetic modification. LncRNA EPB41L4A-AS1 is involved in both glycolysis and glutaminolysis, and its low expression correlates with poor clinical prognosis. EPB41L4A-AS1 predominantly enhances the interaction between HDAC2 and NPM1 in nucleolus, which prevents HDAC2 nucleoplasm translocation and subsequent occupation on VHL and VDAC1 promoters. Therefore, VHL and VDAC1 transcription was increased via histone acetylation, resulting in reduced HIF-1α protein levels and inactivated downstream p-eIF2α/ATF4 pathway, respectively. As HIF-1α could transcriptionally upregulate glycolytic genes and ATF4 activates the expression of amino acid transporters, EPB41L4A-AS1 eventually inhibits glycolysis and glutaminolysis in cancer cells. Moreover, TIGA1, a small protein generated by EPB41L4A-AS1, locates on mitochondrial outer membrane and mediates the connection between α-tubulin and mitochondria. Knockdown of TIGA1 promotes microtubule depolymerization via reduced acetylated α-tubulin, which induces cellular stress and ROS production due to VDAC block by binding with free tubulin. Consequently, p38 is phosphorylated and subsequently increases HIF-1α level 101.

To sum up, lncRNAs function to regulate glutamine metabolism at transcriptional or post-transcriptional level through different mechanisms, and the various properties of lncRNAs contribute to the complexity and multifaceted regulation of glutamine metabolism.

## LncRNAs regulate lipid metabolism

Reprogrammed lipid metabolism in various tumors plays an oncogenic role in multiple aspects of tumorigenesis, including metastasis, interaction with tumor microenvironment, redox stress balance, drug resistance, energy supply, and homeostasis maintenance 102-104. Lipids provide carbon derived from glucose and glutamine for basic cellular framework construction. Moreover, lipids produce energy in nutrient-deficient microenvironment and help modulate redox status by NADPH, one of the consumables in lipid synthesis pathway 104 (Fig. 2).

Sterol regulatory element binding proteins (SREBPs) are key transcription factors that regulate genes for lipid synthesis or lipid transportation in response to fluctuant intracellular lipid level 105. Induced upon HCV infection, lncHR1 suppresses SREBP-1c expression and subsequent fatty acid synthase (FAS) activity, thereby decreasing the abundance of oleic acid-induced triglyceride (TG) and lipid droplet (LD) in hepatoma cells 106. Peroxisome proliferator-activated receptor α (PPARα) is another transcription factor that regulates genes involved in lipid metabolism and its expression is implicated in inflammation, fatty acid oxidation, tumorigenesis, and drug resistance 107. LncRNA nuclear paraspeckle assembly transcript 1 (NEAT1) interferes HCC lipolysis and promotes HCC growth *in vitro* and *in vivo* by transcriptionally maintaining high levels of adipose triglyceride lipase (ATGL) and its catalytic products, diacylglycerol (DAG) and free fatty acid (FFA). In addition, NEAT1 increases fatty acid oxidation (FAO) via the activation of PPARα signaling pathway directly by ATGL or indirectly by DAG and FFA, which is attenuated by transcriptional inhibition of ATGL by miR-124-3p, a downstream effector of NEAT1. Moreover, TP53 positively targets NEAT1 and in turn TP53 expression is suppressed by NEAT1 in HCC 108. However, in other cancer types, NEAT1 is highly expressed independent of p53 or is downregulated by p53 109, 110.

In addition to the regulation of pivotal lipid transcription factors, lncRNAs also regulate lipid metabolism through other mechanisms. For example, linc- adipogenesis and lipogenesis (ADAL) interacts with hnRNPU in the nucleus and IGF2BP2 in the cytoplasm, affects a subset of lipogenic gene expression at transcriptional or post-transcriptional level and regulates pre-adipocyte differentiation as well as de novo fatty-acid biosynthesis in mature adipocytes, thereby altering lipid storage and fatty acid oxidation 111. Acetyl-CoA synthase long-chain 1 (ACSL1) promotes fatty acids uptake and catalyzes the first step in long-chain FA synthesis pathway. One study showed lncRNA HUCL depresses miR-9 targeted PPARα inhibition, then activates expression of ACSL1 and leads to accumulation of cholesterol and triglycerides in hepatoma 112. Another study showed that lncRNA LNMICC, which is negatively regulated by miR-190, recruits transcription factor NPM1 to the promoter of fatty acid binding protein 5 (FABP5) in the nucleus to enhance fatty-acid metabolism. Furthermore, LNMICC correlates with lymph node metastasis, epithelial-mesenchymal transition and lymphangiogenesis in cervical cancer 113.

## LncRNAs regulate tumor microenvironment

Besides synthesized intracellularly, lncRNAs are also exported out of cancer cells or cancer-associated fibroblasts, capsuled in the exosomes and then incorporated by adjacent cells, thus setting up an intercellular signaling pathway and altering tumor microenvironment to modulate tumor metabolism 114, 115.

**Figure 3:**
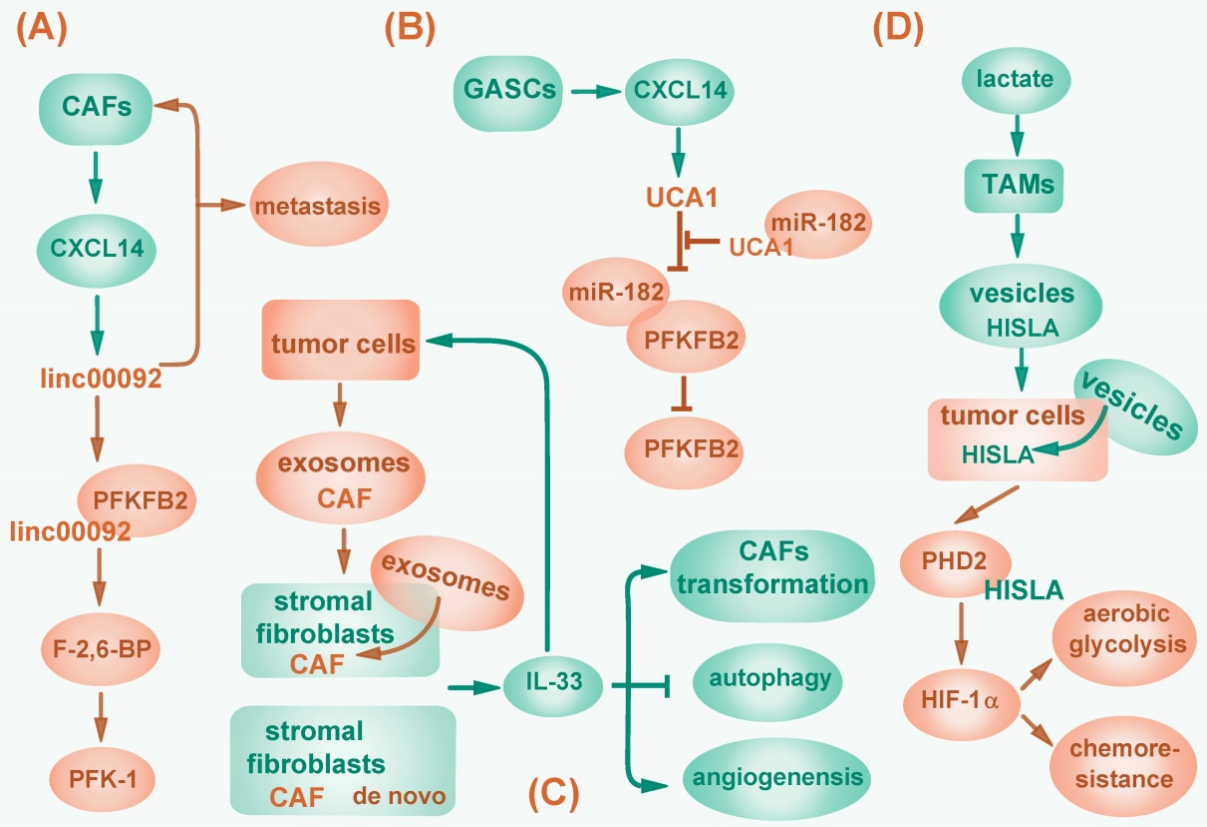
Regulation of cancer-associated fibroblasts by lncRNAs. (A) CXCL14 secreted by CAFs upregulates LINC00092, which binds to PFKFB2 to activate PFK-1 via PFKFB2 catalytic product F-2, 6-BP and then promotes glycolysis. LINC00092 also maintains CAFs-like phenotype and mediates cancer metastasis. (B) CXCL14 secreted by GASCs elevates lncRNA UCA1 expression, then sponges miR-182 to activate PFKFB2. (C) LncRNA-CAF is synthesized in stromal fibroblasts or imported from tumor cell-excreted exosomes to upregulate IL-33, which promotes tumor cell metastasis, mediates CAFs transformation, inhibits CAFs autophagy and induces angiogenesis. (D) Upon lactate stimulation, TAMs synthesize and secret lncRNA HISLA wrapped in vesicles, which are then internalized by cancer cells and abolish HIF-1α degradation by competitively binding to PHD2, consequently promotes glycolysis and chemoresistance.

In addition to function in an autocrine manner, lncRNAs can be regulated by stromal cells in tumor environment. Cancer-associated fibroblasts (CAFs) are a group of tumor stromal mensenchymal cells exhibiting common hallmarks but possessing heterogeneous properties and functions. Moreover, CAFs correlate with tumor initiation, progression and metastasis by modulating tumor microenvironment, and providing nutrients and chemokines via paracrine signaling pathway 116. For example, CXCL14, a pro-metastatic chemokine secreted by CAFs, promotes ovarian cell invasion by upregulating a long intergenic RNA LINC00092 in ovarian cancer cells. LINC00092 enhances glycolysis in ovarian cancer by binding to glycolytic enzyme fructose-2, 6-biphosphatase (PFKFB2) and activating 6-phosphofructo-1-kinase (PFK-1) via PFKFB2 catalytic product fructose-2, 6-bisphosphate (F-2, 6-BP). In turn, CXCL14 maintains the CAFs-like phenotype, thus forming a positive feedback circuit and playing a critical role in cancer metastasis 117 (Fig. 3A). In addition, CXCL14 is also excreted by glioblastoma-associated stromal cells (GASCs) and elevates lncRNA UCA1 expression in cancer cells to activate PFKFB2 by sequestering miR-182 from binding to PFKFB2 mRNA 118 (Fig. 3B). Conversely, lncRNAs can contribute to the aberrant reprogramming in CAFs. One study showed that lncRNA-CAF (Lnc-CAF), synthesized in stromal fibroblasts or internalized from tumor cell-excreted exosomes, specifically mediates the transformation of normal fibroblasts into CAFs in oral squamous cell carcinoma (OSCC) by upregulating IL-33, a chemokine previously reported to reduce apoptosis, promote metastasis, and enable escape from immune system in tumors 119, 120. Lnc-CAF prevents IL-33 degradation through p62-mediated autophagy-lysosome pathway, thus inhibits CAFs autophagy and induces angiogenesis. As a result, lncRNA-CAF supports tumor growth in a positive feedback manner 119 (Fig. 3C). However, whether lnc-CAF induced fibroblast transition correlates with metabolic reprogramming awaits further investigation.

Besides, lncRNAs can induce metabolic reprogramming by mediating interaction between tumor cells and tumor-associated macrophages (TAMs). In breast cancer, upon lactate stimulation, TAMs synthesize and secret HIF-1α-stabilizing long noncoding RNA (HISLA) wrapped in extracellular vesicles. HISLA can be internalized by breast cancer cells and abolishes HIF-1α degradation by competitively binding to PHD2 and interfering HIF-1α hydroxylation, thereby promoting aerobic glycolysis and increasing chemoresistance of tumor cells, which correlates with poor patient prognosis 115 (Fig. 3D).

Taken together, both intracellularly and extracellularly derived-lncRNAs regulate metabolism, consequently, they impact the behavior of cancer cells and modulate tumor microenvironment.

## Perspectives and challenges of lncRNAs in cancer metabolism

So far, lncRNAs have been studied as candidates of cancer molecular biomarkers, contributors to drug resistance and disease progression, as well as potential drug targets along the metabolic pathway. For instance, locked nucleic acid (LNA)-modified antisense oligonucleotides (GapmeRs) could specifically silence SAMMSON and abolish drug resistance to BRAF (V600E) inhibitor dabrafenib and MEK inhibitor pimasertib 121. Liposomal carrier DOPC nanoparticle with incorporated siRNA was also applied to alleviate NRCP-promoted glycolysis in ovarian cancer 68. Different from protein-coding genes, which are mainly monitored based on protein expression in the circulating systems or in the pathological tissues, lncRNAs are detected by PCR with high sensitivity, implying their distinct and possibly more broad applications, particularly in differentiating rare diseases at early stages. However, the development of lncRNA-based clinical applications encounters several obstacles and challenges. For example, although quantities of researches are focusing on the function of lncRNAs in cancer, the intricate mechanisms involved in cancer metabolism require further investigations as only a small fraction of lncRNAs are well elucidated and documented. Moreover, inhibition of oncogenic lncRNAs by siRNA-based approach needs efficient delivering system such as biomolecular material packed nanoparticles. Further optimizations are needed to enable these nanoparticles act precisely on tumor cells. Lastly, some lncRNAs regulate cellular metabolism in non-cancer diseases, such as Tug1 in diabetic nephropathy 49 or Pvt1 in skeletal muscle 122. It will be interesting to study these lncRNAs in tumor models. It is worth noting that the progression in developing diverse analytical methods would enable efficient screening of differentially expressed lncRNAs in cancer and help excavating the underlying mechanisms in cancer metabolism, which hopefully could offer access to clinical applications.

Dysregulated cancer metabolism, a long-discovered phenomenon, is crucial in altered behavior of cancer cells. LncRNAs exert an extensive and complex influence on cancer metabolic effectors and pathways either directly or indirectly, which expands our understanding towards the scope of cancer metabolic reprogramming. Therefore, digging into the functions and mechanisms of lncRNAs in cancer metabolism will be helpful for the identification of new biomarkers and therapeutic targets for cancer treatment.
